# Why is it important to involve people with lived experience in the development of the educational programs in suicide prevention?

**DOI:** 10.1192/j.eurpsy.2021.187

**Published:** 2021-08-13

**Authors:** M. Stensmark

**Affiliations:** Spes Blekinge, SPES, Karlskrona, Sweden

**Keywords:** Lived experience, Grief, Suicide postvention, Family support

## Abstract

Blekinge Family Postvention is a grief facilitator-home-visiting postvention giving early support to families after a suicidal loss since 2015. It helps families overcome shock, pain, anger, guilt, suffering and other extreme emotions caused by suicide. Such situations should be treated not as a disorder. Medication should be used only as a last resort to treat individuals not responding to early family support, followed by individual sessions if needed. Everyone needs to express feelings and thoughts on all aspects of the suddenly interrupted relation. To achieve this, a person experienced in those issues must be leading all of the meetings. The conversations should start during the first days, frequently continuing for at least 3-5 weeks depending on the family needs. Helping the family bear each other and find coping strategies ease their pain, mourning and give them hope instead of prolonged grief. In this way, the family gradually finds new ways to overcome a never-ending negative looping that eventually can cause, e.g. post-traumatic stress disorder and depression. The support to the family after suicide loss should be a governmental matter. Today all work is done voluntarily. The close relatives need debriefing right after the district doctor has stated the death or the police informed the family about suicide. The military, the police, rescue services and healthcare professionals gets debriefing when a rescue operation has failed. This presentation discusses how to organise early family support and the Ellipse project’s interviews with survivors about their experiences of needed or received support if any.

**Disclosure:**

DISCLAIMER The E-Lifelong Learning In Prevention of Suicide In Europe (ELLIPSE)-project is co-funded by the European Union’s Programme Erasmus+ (Project ID: 2019-1-SE01-KA203-060571). The EU Commission’s support for this project does not mean that the Com

